# Motion Object Detection Model for Electronic Referee Scoring in Table Tennis Events

**DOI:** 10.1371/journal.pone.0319558

**Published:** 2025-03-19

**Authors:** Xiaoke Li, Lili Guo

**Affiliations:** Faculty of Physical Education, Pingdingshan University, Pingdingshan, China; Universiti Malaysia Terengganu, MALAYSIA

## Abstract

As a sport widely played around the world, the fairness and enjoyment of table tennis competitions have received increasing attention. Traditional table tennis referees rely on manual judgment, which has problems such as strong subjectivity and high misjudgment rate. Therefore, this study combines the background subtraction method and the Kalman filtering algorithm. It processes missing images in videos to propose a motion object detection and motion estimation model for table tennis events. The test results showed that the average loss value of the model was only 0.33, the average detection accuracy in the 20-category data set was 0.94, and the average detection time was 103.9 ms. In the simulation test, the model achieved the best trajectory prediction accuracy in both complete video images and partially missing information video images. The maximum difference in horizontal and vertical directions was 10.7 and 4.3 pixels, respectively, and the maximum error in three-dimensional coordinates was (3.3, 2.8, 2.1). The table tennis target detection and motion estimation model has high detection accuracy and stability, providing new ideas and methods for the development of electronic referee systems in table tennis competitions.

## 1. Introduction

Nowadays, table tennis is garnering increasing attention due to its perceived fairness and ornamental value [1]. Traditional table tennis referees rely on manual judgment, which has problems such as strong subjectivity and high misjudgment rate, affecting the fairness and accuracy of the game [2]. Therefore, developing automated electronic referee systems has become an important means to improve the fairness of matches. The development of electronic scoring systems for table tennis events is attributed to the rapid advancement of Computer Vision (CV) [3]. CV technology can accurately detect and analyze table tennis trajectory through image processing and pattern recognition technology, while artificial intelligence technology can improve the intelligence level and detection accuracy of the system through deep learning algorithms [4–5].

As for table tennis target detection, various innovative models has been suggested to address the shortcomings and challenges of existing technologies. W. Ren proposed a new method for detecting and recognizing incorrect actions of players using graph CNNs. This method had high accuracy in complex and occluded scenes, with an accuracy of 79.1% on the COCO dataset [6]. Shi Z et al. proposed a table tennis player skill recognition and evaluation method. The recognition accuracy of this method was improved by 6.5% compared with traditional CNNs [7]. Yen C T et al. proposed four basic table tennis hitting classification systems with CNN. The designed sensor and multi-scale CNN performed well in ball recognition [8]. Wang J et al. proposed a visualization analysis system called Tac Miner. The system supported effective analysis and comparison of tactics for multiple matches through advanced embedding and dimensionality reduction algorithms, and interactive symbols [9]. Wang L et al. employed data mining techniques to assess table tennis skills and tactics. The MySQL database was utilized to mark matches, and a table tennis skills and tactics analysis system was constructed [4].

In addition, many scholars have proposed various methods for motion target detection in electronic referee scoring for table tennis events to address the shortcomings of existing technologies. Abulwafa A E et al. proposed a fuzzy-based ball detection strategy for recognizing spheres in different types of sports images. This strategy could still accurately identify balls in occlusion and complex backgrounds [10]. Hashmi M F et al. proposed a method to address the challenge of ball detection in table tennis, while utilizing super-resolution technology to enhance frame resolution. The accuracy of this method for ball position detection was 97.8%, with an F1 value of 98.1%. The accuracy in ball position detection and competition event classification reached 97.8% and 97.47%, respectively [11]. Yang Y et al. proposed a ball detection and trajectory prediction algorithm with CNN and stereo vision. The detection accuracy of neural networks in actual tennis images was 81.4%. The average trajectory prediction errors on the x-axis, y-axis, and z-axis were 29.6 cm, 7.2 cm, and 11.7 cm, respectively [12]. Cruz N et al. proposed a vision system based on CNNs. The system’s robot and ball detection accuracies reached 94.90% and 97.10%, respectively. When observing the robot at rest, the accuracy of the robot direction was 99.88%, and the accuracy during robot motion was 95.52% [13]. S. Siratanita et al. built a model for detecting offside in football matches using multiple cameras. In the video clip experiment of 381 Premier League matches from 2016 to 2017, the detection accuracy of this method reached 98.50% [14].

In addition, different scholars have proposed innovative solutions using image processing, object detection, and deep learning models to improve the robustness, detection accuracy, and adaptability of the system. Khan W et al. proposed a dial reading method based on convolution method. This method was applied to simulated aircraft instruments in the Flight Guardian project to achieve automatic reading of cockpit equipment in dynamic environments [15]. Faisal M M et al. proposed a target detection system based on YOLO V3. Through steering angle measurement and object detection functions, a warning was issued when the car approached pedestrians or other vehicles. The distance of the detected object was calculated by its height. The system achieved good results under low-cost conditions and could be used at night and in low-light environments [16]^.^ Yar H et al. proposed an improved YOLOv5s model for early fire detection. The model integrated the Stem module and improved the SPP and P6 modules to reduce the complexity and size of the model. In addition, a medium-sized fire dataset covering vehicle, building and indoor fires was created, and 12 state-of-the-art detection models were evaluated [17].

To solve the missed detection, insufficient adaptability to complex scenes and trajectory prediction in table tennis target detection, different scholars have proposed innovative methods based on deep learning, fractal artificial intelligence and multi-scale feature fusion. Zhao Y et al. proposed a visual detection method that integrated the frame sorting module and YOLOv5. The frame sorting module used the frame difference method and extended Kalman filter to detect and locate unrecognized images. The results showed that the detection accuracy of this method in the video sequence task reached 94.6% [18]. Li H et al. proposed a table tennis assistance system based on fractal artificial intelligence. The system used structured output convolutional neural network for target tracking. The long short-term memory network and hybrid density network were combined to achieve trajectory prediction [19]. Rong Z proposed a detection method based on deep learning and multi-scale feature fusion. The method combined regional convolutional neural network and multi-level feature information fusion technology to optimize detection performance. Experiments showed that the average detection accuracy of this method reached 87.3% [20].

As an important technical means for table tennis events, the electronic referee system requires the moving target detection model to have high accuracy and real-time performance in complex dynamic backgrounds, partial image information loss, and high-speed moving target scenes. Although the above studies have made certain progress in target detection, trajectory prediction, and dynamic background adaptability, there are still research gaps in the following aspects. First, the detection stability is insufficient. Faced with high-speed motion and complex dynamic backgrounds, the detection accuracy is not stable enough. Second, the processing capability of partial information loss is insufficient. Most studies have failed to effectively address image information loss or occlusion. Finally, the real-time and adaptability are insufficient. While ensuring detection accuracy, existing methods often cannot meet the needs of real-time applications, especially in resource-limited environments.

In view of this, this study proposes a table tennis motion target detection and estimation algorithm with Background Subtraction and Kalman Filter (BS-EKF). A motion target detection and estimation model was designed, and a method for filling missing information in table tennis target images was developed. This model combines Background Subtraction (BS) method and Kalman Filtering (KF), and achieves high-precision table tennis trajectory detection and competition event classification through data fusion and analysis.

This article proposes a motion object detection and estimation method based on BS-EKF, which improves the technical level through the following innovations. First, BS improves the robustness of target detection in dynamic background and illumination change scenes. Experimental results show that after removing BS, the mAP of the model drops from 0.94 to 0.89, which verifies the important role of this method in improving detection accuracy. Secondly, the extended Kalman filter is used to solve the nonlinear trajectory estimation problem of table tennis targets, especially when image information is lost. Compared with KF, EKF improves the trajectory prediction accuracy, and the maximum error in the horizontal and vertical directions is reduced from 17.2 pixels to 10.7 pixels. Finally, the real-time performance is guaranteed by optimizing the algorithm complexity. Experiments show that the BS-EKF model achieves a good balance between accuracy and real-time performance, and its processing time is 103.9 ms, which is suitable for practical applications. Compared with other comparison models, BS-EKF has obvious advantages in accuracy and can meet the real-time processing requirements, proving its application potential in table tennis electronic referee systems.

The core contributions of the research are as follows. (1) A feasible high-precision moving target detection method is proposed under dynamic background, which effectively solves the false detection and missing detection in complex scenes. (2) The extended Kalman filter algorithm is integrated in trajectory prediction, which significantly reduces the prediction error. (3) Through comprehensive experimental verification, it proves that BS-EKF model has significant advantages in detection accuracy, stability and application potential.

The research structure is divided into four parts. The first part reviews the research on moving object detection and electronic judging system. In the second part, an algorithm combining the background difference method and extended Kalman filter is proposed to detect and estimate the moving target of table tennis. The third part analyzes the experimental results of table tennis trajectory prediction based on the improved BS-EKF algorithm. The last part is the research summary and future research direction.

## 2. Methods and materials

### 2.1 Object detection and motion estimation model construction

**Fig 1:**
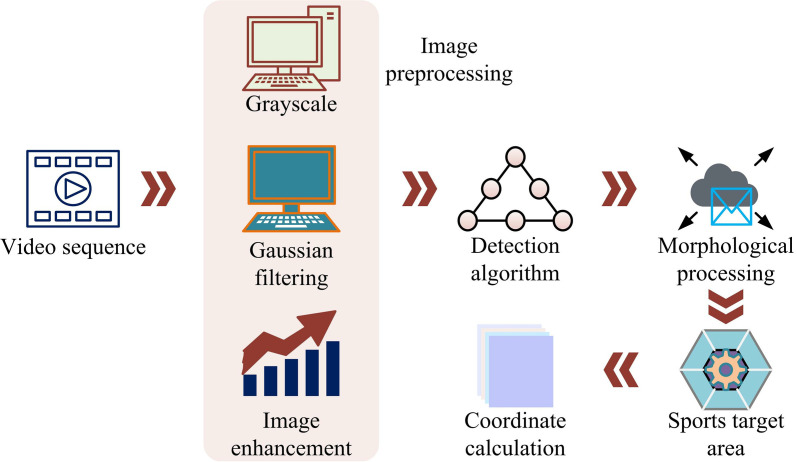
Table Tennis Testing Process Diagram.

Currently, the automatic scoring system for table tennis is a hot research topic. How to accurately and real-time detect the trajectory of table tennis and score it is the core challenge and key link in implementing this system [21]. The complete motion target detection system process is illustrated in Fig 1 [22].

**Fig 2:**
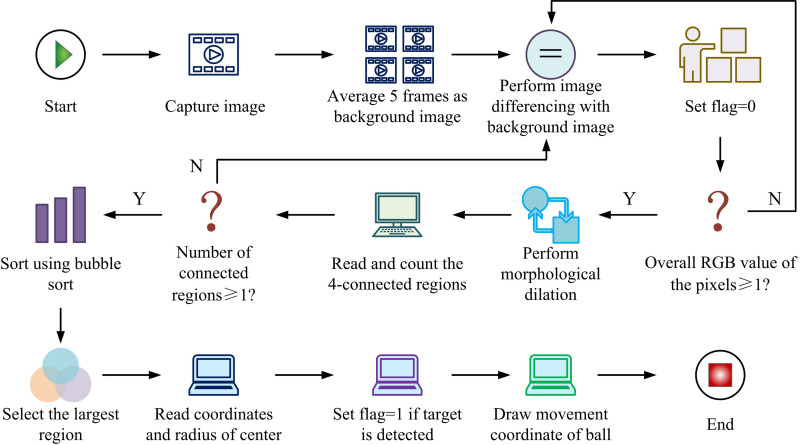
Target Detection Flowchart.

In Fig 1, the system first obtains images from the video sequence. It performs preprocessingg, which includes grayscale, Gaussian filtering, and image enhancement to remove noise and interference from the images while improving image quality. Subsequently, appropriate detection algorithms are employed to analyze the preprocessed images and extract the moving target regions through foreground region processing techniques. The coordinates of the target area of the movement are further calculated to complete the positioning of the table tennis ball. In the detection algorithm stage, the foreground area is refined through morphological processing to remove interference and preserve the target area. Finally, the position of the table tennis ball is calculated and located through coordinates. In terms of algorithm selection, the BS method detects moving targets. This simplifies the computational complexity, meeting the requirements of rapid changes in table tennis [23,24]. Therefore, the BS method is chosen as the main motion detection method. The target detection process based on BS is shown in Fig 2.

In Fig 2, the image is first captured, and the background image is generated by averaging five frames. Secondly, the current frame image is read, its difference from the background image is calculated, and whether the Red Green Blue (RGB) difference of the set threshold image is greater than 10 is determined. If not met, Flag is set to 0 and restart the image acquisition process. Subsequently, if the RGB difference of the image is greater than 10, the image is subjected to morphological denoising. Next, the four connected regions are read and the number of connected regions is calculated. It is determined whether the number of connected regions is not less than 1. If not met, the Flag is set to 0, and the image acquisition process is restarted. Finally, the coordinate map of table tennis is drawn to complete the entire detection process.

**Fig 3:**
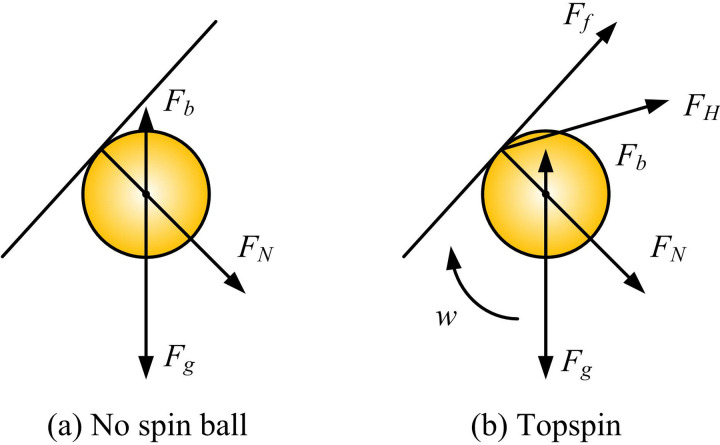
Force Analysis of Irrotational Ball and Topspin.

In selecting motion estimation and tracking algorithms, KF is adopted. KF has excellent dynamic performance and can provide optimal state estimation under noise interference and uncertainty conditions, which makes it suitable for trajectory prediction and position estimation of high-speed moving targets such as table tennis [25,26]. Firstly, it is required to model the motion estimation of table tennis to ensure the accuracy of KF. The force exerted on the topspin of a table tennis ball is shown in Fig 3.

**Fig 4:**
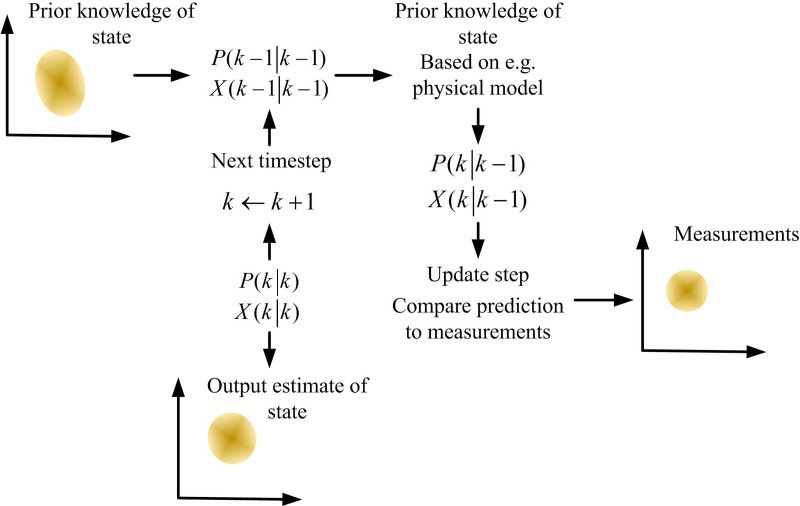
KF Process.

Fig 3 shows the force analysis of the non-spinning and topspin during the tapping process of table tennis. In a non-spinning ball, the ball is only subject to gravity, elasticity, and friction, and the sphere does not rotate. In topspin, the ball is subjected to gravity, elasticity, friction, and Magnus force, and the combined force of these forces causes the ball to rotate. Because this study focuses on high-speed table tennis that is currently in motion during a competition, KF is adopted for motion estimation. KF is a recursive algorithm that can update state estimation in real-time when new data arrives. It can be combined with physical motion models to accurately reflect the motion characteristics of table tennis through the setting of the state transition matrix and observation matrix. The working process of KF is shown in Fig 4 [27].

In Fig 4, in the prediction stage, the current state is first predicted through the state transition equation, and the predicted state vector is calculated. Secondly, the predicted state covariance is calculated. In the calibration phase, the Kalman gain is first calculated. Then, the observed values are used to correct the predicted values, and the state estimation is updated. Finally, the error covariance is updated to reflect the uncertainty of the new state estimation. Therefore, the state expression of the target motion is shown in Equation (1) [28].


$$X(k |k-1)=A(k)X(k-1/k-1)+W(k-1)$$ (1)

In Equation (1), $X(k |k-1)$ denotes the predicted state vector at time *k*. A(k) denotes the state transition matrix. X(k−1/k−1) denotes the estimated state vector at time k−1. W(k−1) represents the process noise vector. Subsequently, the expression for the target motion observation is shown in Equation (2) [29].


Z(k)=H(k)X(k−1)+V(k−1) (2)

In Equation (2), Z(k) denotes the observation vector at time *k*. H(k) denotes the observation matrix. V(k−1) denotes the observation noise vector. Therefore, the state definition of the motion model in the competition video is shown in Equation (3) [30].


$$X(k)=[\begin{array}{cccc} p_{k}(x) & p_{k}(z) & v_{k}(x) & v_{k}(x) \end{array}]$$ (3)

In Equation (3), $p_{k}(x)$ and $p_{k}(z)$ respectively represent the displacement of the object on the X and Z axes at time *k*. $v_{k}(x)$ and $v_{k}(x)$ respectively denote the velocity of the object on the X and Y axes at time *k*. Subsequently, the initial values predicted by Kalman are shown in Equations (4) and (5) [31].


$$\left[\begin{array}{c} v_{x}(2)\\ v_{z}(2) \end{array}\right]=\frac{1}{\Delta t}\left(\left[\begin{array}{c} p_{x}(2)\\ p_{z}(2) \end{array}\right]-\left[\begin{array}{c} p_{x}(1)\\ p_{z}(1) \end{array}\right]\right)$$ (4)

$$a_{x}(2)=k_{f}V(2)V_{x}(2)$$ (5)

In Equations (4) and (5), $v_{x}(2)$ and $v_{z}(2)$ represent the velocities of time $t_{2}$ on the X and Z axes, respectively. Δt represents the time interval. $p_{x}(2)$ and $p_{z}(2)$ represents the position of time $t_{2}$ on the X and Z axes, respectively. $p_{x}(1)$ and $p_{z}(1)$ represent the position of time $t_{1}$ on the X and Z axes, respectively. $a_{x}(2)$ represents the acceleration of time $t_{2}$ in the X axis. $k_{f}$ represents the air resistance coefficient. V(2) represents the speed of time $t_{2}$. $V_{x}(2)$ represents the velocity of time $t_{2}$ in the X direction. Due to the non-linear nature of trajectory estimation for spinning table tennis balls, this study selects the Extended Kalman Filter (EKF) for problem transformation. EKF is an extended algorithm used for processing state estimation of nonlinear systems. Unlike standard Kalman filters, EKF can linearize the state transitions and observation equations of nonlinear systems, making it suitable for a wider range of application scenarios [32]. The prediction expression is shown in Equations (6), (7) and (8) [33].


$$X(k |k-1)=F(X_{k-1},U_{k-1})$$ (6)

$$P(k |k-1)=A_{k-1}P(k-1)A_{k-1}{}^{T}+Q_{k}$$ (7)

$$A_{k}=\frac{\partial F}{\partial X}(X_{k-1},U_{k-1})$$ (8)

In Equations (6), (7) and (8), $F(X_{k-1},U_{k-1})$ represents the state transition function. $P(k |k-1)$ represents the predicted state covariance matrix at time *k*. $A_{k}$ and $A_{k-1}$ respectively represent the Jacobian matrix at time *k* and k−1. P(k−1) denotes the state covariance matrix at time k−1. $Q_{k}$ represents the process noise covariance matrix. $\frac{\partial F}{\partial X}$ represents the state transition function *F* ‘s partial derivative of the state vector *X*. To more accurately predict the trajectory of a table tennis ball in three-dimensional space, this study proposes an EKF algorithm that combines the characteristics of table tennis motion. It includes the definition of state variables, state transition models, observation models, and methods for adjusting process and observation noise, and gradually establishes EKF improvement logic. First, in the state variable definition, the dynamic characteristics of table tennis motion require a multidimensional state vector containing position, velocity, and angular velocity of rotation to comprehensively describe the trajectory change. The state vector is defined as Equation (9).


$${\text{x}}_{k}={\left[\begin{array}{ccccccc} x_{k} & y_{k} & z_{k} & v_{x_{k}} & v_{y_{k}} & v_{z_{k}} & w_{k} \end{array}\right]}^{T}$$ (9)

In Equation (9), $x_{k}$, $y_{k}$, and $z_{k}$ represent the three-dimensional spatial position of the ping-pong ball at the current time step. $v_{x_{k}}$, $v_{y_{k}}$, and $v_{z_{k}}$ represent the velocity components of the ping-pong ball in the three coordinate directions. $w_{k}$ is the angular velocity of the ping-pong ball. By defining the state vector, the EKF can simultaneously track the nonlinear dynamic characteristics of the spatial position and trajectory changes of the ping-pong ball. In the state transition model, to describe the motion process of the ping-pong ball, the state transition model combines the gravity, air resistance, and Magnus force on the ping-pong ball. The differential form of the state change is shown in Equation (10).


$$\dot{\text{x}}\text{=}\left[\begin{array}{c} {\dot{x}}_{k}\\ {\dot{y}}_{k}\\ {\dot{z}}_{k}\\ {\dot{v}}_{x_{k}}\\ {\dot{v}}_{y_{k}}\\ {\dot{v}}_{z_{k}}\\ {\dot{w}}_{k} \end{array}\right]=\left[\begin{array}{c} v_{x_{k}}\\ v_{y_{k}}\\ v_{z_{k}}\\ -\frac{1}{2}C_{d}A\rho v_{x_{k}}^{2}/m+F_{M}(x,w)\\ -g-\frac{1}{2}C_{d}A\rho v_{y_{k}}^{2}/m+F_{M}(y,w)\\ -\frac{1}{2}C_{d}A\rho v_{z_{k}}^{2}/m+F_{M}(z,w)\\ -\beta \cdot w_{k} \end{array}\right]$$ (10)

In Equation (10), $F_{M}(x,w)$, $F_{M}(y,w)$, and $F_{M}(z,w)$ are the Magnus force components. In the observation model, the observation data of the table tennis ball is obtained through the video frame, mainly including the position information. The observation model assumes that the observation value is part of the state vector. The expression with observation noise is shown in Equation (11).


$$z_{k}=\text{H}\cdot {\text{x}}_{k}+{\text{w}}_{k}$$ (11)

In Equation (11), $z_{k}$ is the observation value, $\left[\begin{array}{ccc} x_{k} & y_{k} & z_{k} \end{array}\right]$ is the three-dimensional spatial position, H is the observation matrix, and ${\text{w}}_{k}$ is the observation noise, which obeys the zero-mean Gaussian distribution. Subsequently, in order to adapt to the dynamic background and noise changes, the process noise and observation noise covariance matrix needs to be dynamically updated. The process noise covariance matrix is shown in Equation (12).


$$Q_{k}=diag\left(\left[\sigma_{x}^{2},\sigma_{y}^{2},\sigma_{z}^{2},\sigma_{v_{x}}^{2},\sigma_{v_{y}}^{2},\sigma_{v_{z}}^{2},\sigma_{w}^{2}\right]\right)$$ (12)

In Equation (12), $\sigma_{y}^{2}$, $\sigma_{y}^{2}$, and $\sigma_{z}^{2}$ are the uncertainty variances of position prediction. $\sigma_{v_{x}}^{2}$, $\sigma_{v_{y}}^{2}$, and $\sigma_{v_{z}}^{2}$ are the uncertainty variances of velocity prediction. $\sigma_{w}^{2}$ is the uncertainty variance of rotation angular velocity. Then, the observation noise covariance matrix is shown in Equation (13).


$$R_{k}=diag\left(\left[\sigma_{x_{obs}}^{2},\sigma_{y_{obs}}^{2},\sigma_{z_{obs}}^{2}\right]\right)$$ (13)

In Equation (13), $\sigma_{x_{obs}}^{2}$, $\sigma_{y_{obs}}^{2}$, and $\sigma_{z_{obs}}^{2}$ are the uncertainty variances of the observed x, y, and z positions, respectively. Therefore, according to the above calculations, the application of the improved EKF for the nonlinear trajectory characteristics in table tennis target detection includes two core stages: prediction and update. First, the next position, speed, and angular velocity of the table tennis ball are predicted by the state transition model, and the error range is estimated. Subsequently, when new observation data is obtained from the video frame, the observation model is used to calculate the residual between the observed value and the predicted value. The state estimate is corrected by the Kalman gain. To adapt to dynamic backgrounds and complex scenes, the process noise and observation noise covariance matrices are dynamically adjusted according to the speed change and image quality, thereby improving the prediction stability and accuracy.

To verify the performance of the improved EKF algorithm in table tennis target detection, this study selects three datasets, including TTNet, PingPongNet, and SportsNet. These datasets cover standard scenes, high dynamic scenes, and complex scenes across sports in table tennis games, with different scales and diversity, which helps to comprehensively evaluate the adaptability and robustness of the algorithm in various environments. Table 1 lists the number of samples and their division methods of each dataset in detail.

**Table 1:** Dataset and Division Information.

Sample Type	TTNet	PingPongNet	SportsNet
Total samples	55582	75000	120000
Training samples	38752	52500	72000
Validation samples	9502	15000	24000
Test samples	7328	7500	24000
Dataset features	High-frame-rate table tennis scenes with standard annotations	High-dynamic scenes with diverse lighting and background conditions	Multi-sport projects with diverse backgrounds and annotations

In Table 1, TTNet is a high-frame-rate table tennis match dataset with precise annotations, which is mainly used to verify the detection accuracy and trajectory prediction ability of the algorithm in standard scenarios. The PingPongNet dataset records table tennis at a higher frame rate and contains complex backgrounds and multiple lighting conditions, which is used to evaluate the stability of the model under dynamic backgrounds and diverse scenarios. The SportsNet dataset covers target detection tasks for multiple sports, especially the table tennis part, which accounts for 40%. Its complexity provides a good foundation for the scalability verification of the model.

### 2.2 Research design on image information deficiency

**Fig 5:**
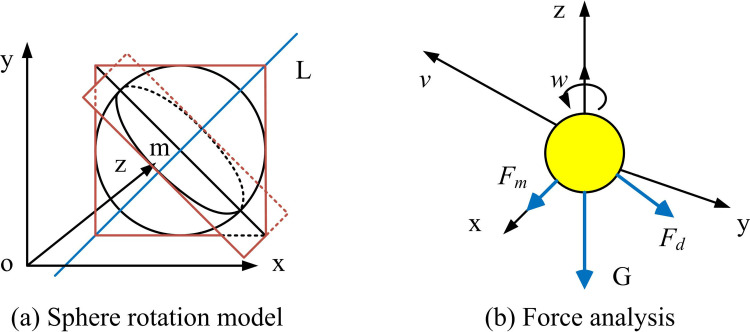
Force Diagram of a Flying Ball.

In practical applications, the problem of missing information in table tennis target images often has a negative influence. Due to the fast speed and varied trajectory of table tennis during the game, there may be blurriness, occlusion, or frame loss in the image capture process, which can lead to the loss of some image information [34]. Therefore, this study start with the force on the target table tennis ball. A corresponding motion model is established. Fig 5 illustrates the force diagram.

In Fig 5, table tennis is subjected to four main forces during flight. They are respectively the gravity *G*, the air resistance $F_{d}$, the buoyancy $F_{b}$, and the Magnus force $F_{m}$. Therefore, the expression of the force on a table tennis during flight is shown in Equations (14), (15), (16) and (17) [35].


$${\vec{F}}_{g}={\left[\begin{array}{ccc} 0 & 0 & -mg \end{array}\right]}^{T}$$ (14)

$${\vec{F}}_{d}=\frac{1}{2}\rho SC_{d}{\vec{V}}^{2}$$ (15)

$${\vec{F}}_{m}=\frac{1}{2}\rho wr_{b}SC_{l}\vec{V}$$ (16)

$${\vec{F}}_{b}={\left[\begin{array}{ccc} 0 & 0 & \frac{1}{6}\pi \rho d^{3}g \end{array}\right]}^{T}$$ (17)

In Equations (14), (15), (16) and (17), ${\vec{F}}_{g}$, ${\vec{F}}_{d}$, ${\vec{F}}_{m}$, and ${\vec{F}}_{b}$ respectively represent gravity, air resistance, Magnus force, and air buoyancy. *m* is the mass. *S* is the windward area of the sphere. $C_{d}$ represents the air resistance coefficient. $\vec{V}$ represents the velocity of the sphere. $r_{b}$ and *d* respectively represent the radius and diameter of the sphere. *g* is the acceleration due to gravity. $C_{l}$ represents the rise factor. Subsequently, Table 2 shows the parameter values in the physical model.

**Table 2:** Parameter Setting in Physical Models.

Parameter	Value
*g*	9.802m/s²
*m*	0.0027 kg
*ρ*	1.29 × 10³kg/m³
*w*	0 ~ (10 × 2π)rad/s
*V*	3 ~ 10m/s
$k_{f}$	0.00035
$r_{b}$	0.02m
$C_{l}$	[0.2,0.6]
$C_{d}$	[0.4,0.5]

In Table 2, the combined force of these forces determines the motion and balance of the ball. Therefore, the buoyancy value is shown in Equation (18).


$$F=\frac{4}{3}\rho g\pi r^{3}=3.8\times 10^{-4}N$$ (18)

In Equation (18), *ρ* is the air density. Due to the significant difference in buoyancy values and the negligible impact, the influence of buoyancy is ignored in the calculation. At the same time, due to the uncertainty of human serving, it is difficult to model the rotational angular velocity. Therefore, Magnus force is also ignored. This study only considers the influence of gravity on the falling speed and deceleration of table tennis balls, as well as the influence of air resistance on the flight trajectory of table tennis balls. Through the above simplification, a mathematical model can be more easily established and analyzed, which is helpful for understanding and predicting the table tennis ball’s flight trajectory. Afterwards, the model became more controllable and accurate. The expression is shown in Equation (19).


$$m\vec{v}={\vec{F}}_{g}+{\vec{F}}_{d}$$ (19)

In Equation (19), $\vec{v}$ represents the velocity vector. Therefore, according to Equation (8), the change law of the motion state of table tennis during flight can be obtained to predict the flight trajectory of table tennis, as shown in Equations (20) and (21).


$$\left[\begin{array}{c} \vec{x}\\ \vec{y}\\ \vec{z} \end{array}\right]=\left[\begin{array}{c} v_{x}\\ v_{y}\\ v_{z} \end{array}\right]$$ (20)

$$\left[\begin{array}{c} {\vec{v}}_{x}\\ {\vec{v}}_{y}\\ {\vec{v}}_{z} \end{array}\right]=\left[\begin{array}{c} -k_{m}v_{x}^{2}\\ -k_{m}v_{y}^{2}\\ -k_{m}v_{z}^{2}-g \end{array}\right]$$ (21)

In Equations (20) and (21), $\vec{x}$, $\vec{y}$, and $\vec{z}$ respectively are the change rate of the position of the table tennis in the X, Y, and Z coordinate directions. $v_{x}$, $v_{y}$, and $v_{z}$ respectively are the acceleration of table tennis in three coordinate directions. ${\vec{v}}_{x}$, ${\vec{v}}_{y}$, and ${\vec{v}}_{z}$ respectively are the velocity components of table tennis in three coordinate directions. $k_{m}$ represents the air resistance coefficient, which is the relationship coefficient between air resistance and the square of velocity. The expression of $k_{m}$ is shown in Equation (22).


$$k_{m}=\frac{1}{2m}\rho SC_{d}$$ (22)

According to Equation (22), the specific correlation between velocity change and position movement at the current time node can be established compared with the previous time node, as expressed in Equations (23) and (24).


$$\left[\begin{array}{c} x_{t}\\ y_{t}\\ z_{t} \end{array}\right]=\left[\begin{array}{c} x_{t-1}\\ y_{t-1}\\ z_{t-1} \end{array}\right]=\left[\begin{array}{c} V_{xt-1}\\ V_{yt-1}\\ V_{zt-1} \end{array}\right]T_{c}$$ (23)

$$\left[\begin{array}{c} V_{xt}\\ V_{yt}\\ V_{zt} \end{array}\right]=\left[\begin{array}{c} V_{xt-1}\\ V_{yt-1}\\ V_{zt-1} \end{array}\right]=\left[\begin{array}{c} -k_{m}\left\|\vec{V_{xt-1}}\right\|V_{xt-1}\\ -k_{m}\left\|\vec{V_{yt-1}}\right\|V_{yt-1}\\ -k_{m}\left\|\vec{V_{zt-1}}\right\|V_{zt-1} \end{array}\right]T_{c}$$ (24)

In Equations (23) and (24), *t*, and t−1 are the current and previous time, respectively. $T_{c}$ represents the time step. $x_{t}$, $y_{t}$, and $z_{t}$ represent the position coordinates at time *t*. $V_{xt-1}$, $V_{yt-1}$, and $V_{zt-1}$ represent the velocity components at time t−1. $\vec{V_{xt-1}}$, $\vec{V_{yt-1}}$, and $\vec{V_{zt-1}}$ represent the modulus of the velocity vector at time t−1. The expression for the velocity change of the ping-pong ball in the X direction due to air resistance $k_{m}$ is shown in Equation (25).


$$m\frac{d_{v_{x}}}{dt}=-k_{f}v_{x}^{3}\begin{array}{cc} , & v_{xo}>0 \end{array}$$ (25)

In Equation (25), $\frac{d_{v_{x}}}{dt}$ represents the time derivative of velocity. $k_{f}$ represents the air resistance coefficient. $v_{xo}$ represents the initial velocity. Therefore, the displacement calculation of the table tennis under air resistance is shown in Equation (26).


$$k=\frac{m}{k_{f}}\ln(1+\frac{k_{f}v_{xo}t}{m})+x_{o}\begin{array}{cc} , & v_{xo}>0 \end{array}$$ (26)

**Fig 6:**
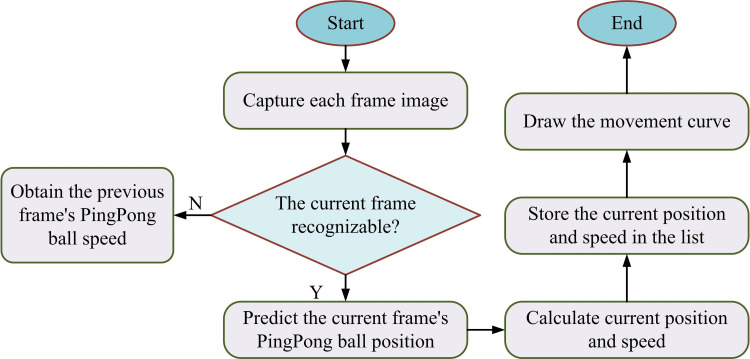
The Process of Processing Images with Missing Targets.

In Equation (26), $x_{o}$ represents the initial position. Therefore, based on the calculated the initial velocity, the displacement and velocity of *t* at the current time can be obtained. According to the above calculation, the processing flow for missing images of moving targets in the video sequence is shown in Fig 6.

In Fig 6, each frame image is first obtained. Subsequently, the velocity of the preceding frame is calculated, thereby estimating the position and velocity in the current frame. These estimated values are utilized to determine the expected position and velocity, and saved to the current frame. Finally, the motion trajectory map is drawn based on the estimated position information.

## 3. Results

A suitable experimental environment is first established and the test data is preprocessed. Secondly, experimental testing is conducted on the improved BS-EKF motion object detection algorithm to evaluate its detection performance on different targets. Subsequently, simulation tests are conducted in practical application scenarios to test the actual effectiveness.

### 3.1 Performance testing of motion target detection

This study uses Windows 10 system, equipped with Intel (R) Xeon (R) Gold 5218R CPU model, 16GB of memory, and NVIDIA GeForce RTX 3060 LapTop GPU 6GB. You Only Look Once version 4 (YOLOv4), Faster Region based Convolutional Neural Network (Faster R-CNN), and Single Shot MultiBox Detector (SSD) are selected as comparison algorithms. These models are representative as the mainstream deep learning methods in the current field of target detection and trajectory prediction. They are widely used in different application scenarios, among which YOLOv4 is known for its real-time performance, Faster R-CNN is known for its high detection accuracy, and SSD has high applicability in multi-target detection. To ensure the fairness and scientificity of the experimental results, YOLOv4, Faster R-CNN and SSD are specially customized for training. The training data comes from the TTNet, PingPongNet, and SportsNet datasets. These three datasets contain rich table tennis target annotation information, covering a variety of lighting conditions, dynamic backgrounds, and occlusion scenarios. During the training process, the model parameters are first initialized using pre-trained weights, and then the model is fine-tuned in the training set to adapt to the specific needs of table tennis target detection. The learning rate of YOLOv4 is set to 0.001, and the number of training rounds is 150. The learning rate of Faster R-CNN is set to 0.0002, with a distributed gradient descent optimizer, and the number of training rounds is 200. The learning rate of SSD is 0.001 and the number of training epochs is 100. Through customized training, these deep learning models can better adapt to the table tennis scene characteristics, allowing for a fairer performance comparison with the proposed BS-EKF method.

First, to verify the effectiveness of the key modules of the proposed BS-EKF model, an ablation experiment is designed to evaluate the model performance from the perspective of basic indicators. The experiment is conducted on the TTNet dataset. The effects of the background difference method, EKF, and dynamic noise adjustment module on the detection accuracy (Mean Average Precision, mAP) and average detection time are investigated. The experimental results are shown in Table 3.

**Table 3:** Ablation test results.

Model Configuration	mAP	F1 score	Average detection time/ms
BS-EKF	0.94	0.93	103.9
Without background subtraction (BS)	0.89	0.86	110.3
Replace EKF with KF	0.91	0.89	101.7
Without Dynamic Noise Adjustment (Fixed Noise)	0.92	0.9	102.8

**Fig 7:**
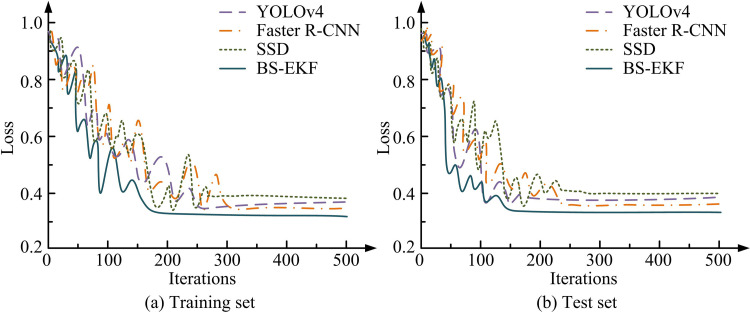
Loss Change Curve Test Results.

In Table 3, the full model BS-EKF achieved the best performance in all indicators, with an mAP of 0.94 and an F1 score of 0.93, and maintained a high detection efficiency of 103.9ms. After removing BS, the detection accuracy and F1 score dropped to 0.89 and 0.86, respectively, and the detection time increased by about 6.4ms, indicating the importance of BS in dealing with dynamic background interference. After replacing the improved EKF with the ordinary KF, both the detection accuracy and F1 score decreased. In addition, after removing the dynamic noise adjustment module, although the detection time was slightly shortened, the detection accuracy and F1 score still decreased. This module improved the adaptability of the model under complex backgrounds. Subsequently, the loss change curves of the four models on the dataset are shown in Fig 7.

**Fig 8:**
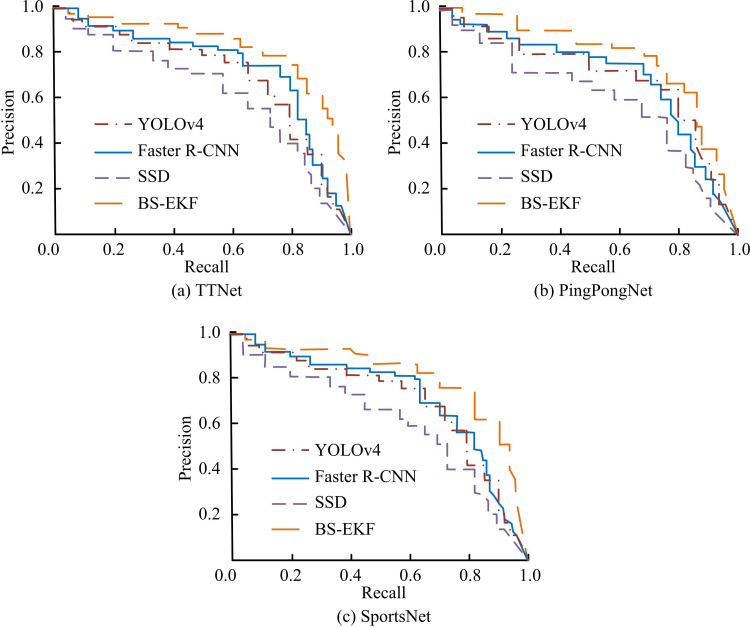
Detection Accuracy and Recall Test Results.

Figs 7(a) and 7(b) show the loss variation curves of YOLOv4, Faster R-CNN, SSD, and BS-EKF models on the training and testing sets, respectively. In Fig 7(a), when the number of iterations was 500, the loss values of YOLOv4, Faster R-CNN, SSD, and BS-EKF were 0.36, 0.35, 0.38, and 0.32, respectively. In Fig 7(b), when the number of iterations was 500, the loss values of the four models were 0.39, 0.36, 0.40, and 0.34, respectively. BS-EKF had the lowest loss values in both the training and testing sets, demonstrating good convergence. Subsequently, the Precision Recall (PR) curves of different models are shown in Fig 8.

**Fig 9:**
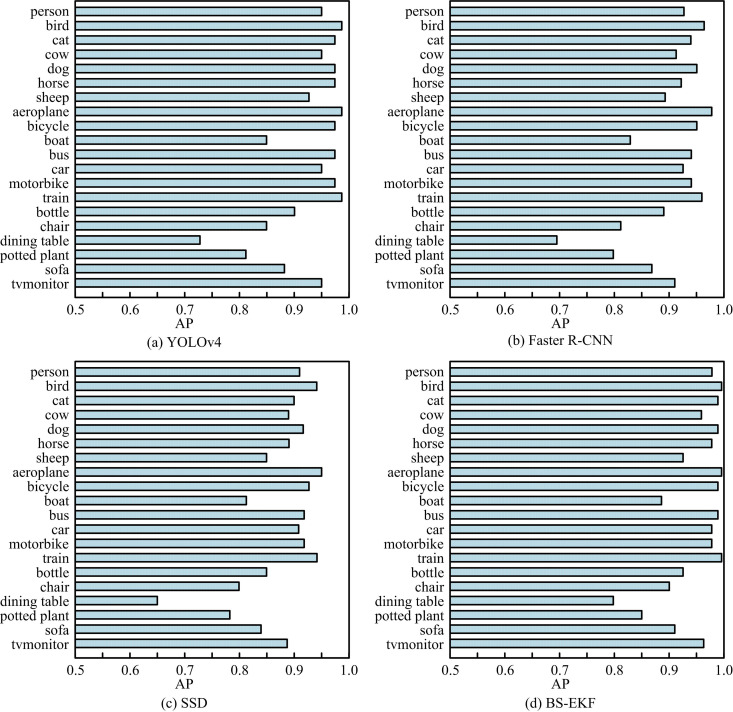
Average Accuracy Test of Four Models.

Figs 8(a) - (c) show the PR curves of four models in the three datasets of TTNet, PingPongNet and SportsNet, respectively. The PR curve of the BS-EKF model was significantly better than other comparison models in all datasets. In the TTNet dataset in Fig 8(a), the BS-EKF model maintained high Precision in the high Recall area, demonstrating its excellent performance in standard scenarios. In Fig 8(b), faced with complex background and illumination changes, the PR curve of the BS-EKF model still fully surrounded other models, indicating its robustness in complex scenes. In the SportsNet dataset shown in Fig 8(c), although the dataset had diverse scenarios and many interference items, the PR curve of the BS-EKF model still remained ahead. Especially in the low Recall area, the Precision was significantly higher than other models. Furthermore, to validate the performance of the model on multi-classification problems, this study uses the benchmark dataset VOC2007 in CV research. This dataset contains 9963 images, covering 20 categories of objects. The mAP results of different models are shown in Fig 9.

In Fig 9(a), YOLOv4 had high detection accuracy for most categories, with an mAP value of 0.93. In Fig 9(b), the mAP value of Faster R-CNN was 0.90, which performed well in multiple categories. In Fig 9(c), the mAP value of SSD was 0.88. SSD performs slightly worse than YOLOv4 and Faster R-CNN in small target detection and complex backgrounds. In Fig 9(d), the mAP value of the BS-EKF model was 0.94, indicating high detection accuracy across all target categories, particularly in small targets and dynamic scenarios. Accurate object detection was achieved through BS method, and the target trajectory was optimized using extended Kalman filter. This model effectively improved detection accuracy and stability. Finally, the detection speeds of the four models for the different categories mentioned above are shown in Table 4.

**Table 4:** Different Types of Detection Time.

Sample category	Model detection speed/ms			
	YOLOv4	Faster R-CNN	SSD	BS-EKF
Person	56.1	241.3	46.5	93.2
Bird	53.4	233.7	42.2	92.3
Cat	64.2	256.6	51.1	106.4
Cow	66.3	261.5	56.7	104.8
Dog	52.8	233.4	44.9	92.2
Horse	61.7	256.8	55.5	111.6
Sheep	56.4	243.2	46.8	114.7
Aeroplane	54.6	236.9	44.5	94.2
Bicycle	64.4	253.3	55.7	110.5
Boat	71.2	276.8	60.4	122.9
Bus	56.9	243.4	46.6	96.3
Car	61.3	253.7	50.8	114.2
Motorbike	56.7	241.6	46.1	112.5
Train	72.6	272.9	62.8	106.6
Bottle	54.9	231.8	42.6	96.4
Chair	66.8	263.3	56.4	111.9
Diningtable	76.5	274.7	63.7	104.8
Pottedplant	56.3	234.6	41.8	91.7
Sofa	56.2	241.9	46.2	106.4
TV monitor	61.5	256.2	50.9	94.4
Average	61.0	250.4	50.6	103.9

Table 4 illustrates the detection speed of four models on different sample categories. YOLOv4 and SSD exhibited faster detection speeds in all categories, with average detection time of 61.0ms and 50.6ms, respectively. The model was suitable for real-time applications. YOLOv4, with its fast and precise features, was slightly faster than SSD. Due to the used two-stage detection method, Faster R-CNN had a slow detection speed, with an average detection time of 250.4ms in different categories, it was not suitable for real-time scenarios. BS-EKF combined BS and EKF, with an average detection speed of 103.9ms and high computational complexity, making it suitable for handling scenarios with complex dynamic backgrounds. Overall, YOLOv4 and SSD had advantages in speed and efficiency, while BS-EKF had more potential in handling complex scenes and improving detection accuracy, without significantly improving runtime while maintaining high detection accuracy.

### 3.2 Motion estimation and prediction simulation experiment testing

**Fig 10:**
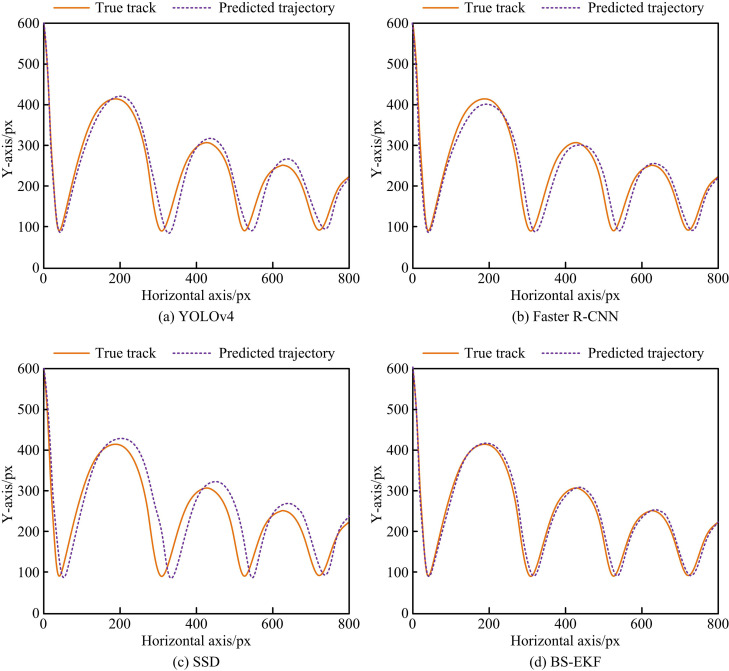
Predicted Trajectory and actual Trajectory Test Results.

After conducting performance tests on BS-EKF, this study further simulates and tests the practical application of the table tennis motion target detection and estimation model. This study randomly selects a table tennis video sequence from the TTNet dataset. The tracking and prediction results of the four models are shown in Fig 10.

**Fig 11:**
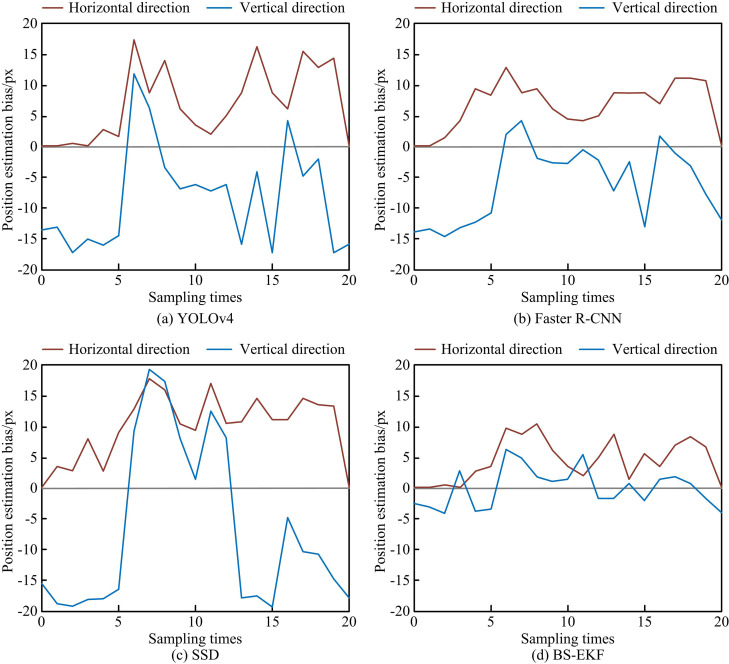
The Difference Between Trajectory Prediction and Measured Value.

Figs 10 (a) - 10(d) illustrate the comparison results of four models in predicted and actual trajectories of table tennis movements. The predicted trajectory trends of each model were relatively consistent, but there were deviations at different landing points. The predicted trajectory deviation of the SSD model in Fig 10(c) was the largest, with significant errors between the highest point of the trajectory and the collision point. In Figures 10 (a) and 10 (b), the predicted trajectories of YOLOv4 and Faster R-CNN models were similar to the actual trajectory test results, with a deviation smaller than SSD. In Fig 10(d), the BS-EKF only had deviation at the falling point, and its predicted trajectory was most consistent with the true trajectory, with a small difference in position. Subsequently, this study expands the experimental scope. The difference in predicting the trajectory of table tennis in the horizontal and vertical directions for 20 experiments using different models is shown in Fig 11.

**Fig 12:**
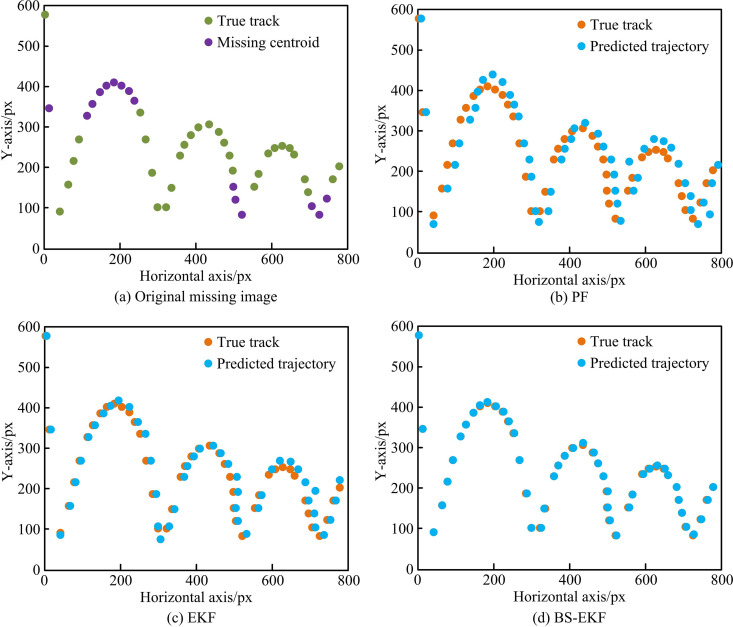
Capped and Valley Target Missing Trajectory Prediction Test.

Fig 11(a) - 11(d) illustrate the position estimation deviation results. In Fig 11(a), the maximum difference between the horizontal and vertical directions of YOLOv4 was 17.2 and 18.7 pixels, respectively. In Fig 11(b), the maximum difference between the horizontal and vertical directions of Faster R-CNN was 13.3 and 14.1 pixels, respectively. In Fig 11(c), the maximum difference between the horizontal and vertical directions of the SSD was 18.1 and 18.8 pixels, respectively. In Fig 11(d), the maximum difference between the horizontal and vertical directions of BS-EKF was 10.7 and 4.3 pixels, respectively. In this test, SSD had the largest estimation bias, followed by YOLOv4, and Faster R-CNN. The Kalman filter model had the smallest estimation bias. The reason is that SSD has significant advantages in speed and efficiency, but its accuracy is slightly inferior to other models, especially in detecting small targets and complex backgrounds. BS-EKF recursively updates and predicts the target position based on the previous state and current observation values. For fast-moving targets like table tennis, the EKF can provide continuous and smooth trajectory estimation with high accuracy and low estimation bias. Subsequently, the study introduces Particle Filter (PF) and EKF as comparative models to predict and estimate the motion trajectory of video images with partially missing moving targets using different models, as shown in Fig 12.

Fig 12(a) shows the trajectory of a table tennis ball with missing original targets. The purple dots in the figure represent the missing parts, while the green dots represent the test image. Figs 12(b) - 12(d) show the target missing processing results of PF, EKF, and BS-EKF and the actual test trajectory. The orange dots represented the actual motion trajectory, and the blue dots represented the predicted trajectory. In Fig 12(b), the predicted trajectory of PF was in good agreement with the true trajectory when dealing with missing trajectories, but deviated significantly at certain points, especially at complex trajectory changes. In Fig 12(c), the predicted trajectory of EKF had a higher overall agreement with the true trajectory, but there was also some deviations when the trajectory changes rapidly. In Fig 12(d), BS-EKF performed the best in handling missing trajectories, with the predicted trajectory almost completely coinciding with the true trajectory, demonstrating high estimation accuracy. The BS-EKF model had significant advantages in addressing the problem of missing target trajectory estimation, and could provide more accurate trajectory prediction results. To visualize the prediction error, the 3D coordinate error information of the 15 missing points is shown in Table 5.

**Table 5:** Error Analysis Between Real 3D Coordinates and Predicted Coordinates.

Serial number	Model		
	PF model error/mm	EKF model error/mm	BS-EKF model error/mm
1	(4.2, 3.0, 1.5)	(2.8, 2.4, 1.2)	(1.5, 1.2, 0.6)
2	(9.0, 5.6, 2.7)	(5.4, 4.6, 2.4)	(0.0, 0.1, 0.3)
3	(5.2, 3.8, 1.8)	(3.6, 2.8, 1.5)	(2.0, 1.6, 0.9)
4	(6.0, 6.6, 2.2)	(4.4, 3.6, 1.9)	(2.5, 2.0, 1.3)
5	(3.4, 2.1, 1.1)	(2.0, 1.8, 0.9)	(1.0, 0.8, 0.4)
6	(11.6, 6.2, 3.0)	(6.0, 5.2, 2.7)	(3.3, 2.8, 2.1)
7	(5.6, 9.2, 2.0)	(4.0, 3.2, 1.7)	(2.3, 1.8, 1.1)
8	(7.8, 6.4, 3.1)	(6.2, 5.4, 2.8)	(3.4, 2.9, 2.2)
9	(9.2, 4.8, 2.3)	(4.6, 3.8, 2.0)	(2.6, 2.1, 1.4)
10	(3.6, 2.8, 1.3)	(2.2, 2.0, 1.0)	(1.1, 1.0, 0.5)
11	(10.2, 5.8, 2.8)	(5.6, 4.8, 2.5)	(3.1, 2.6, 1.9)
12	(5.0, 3.6, 1.7)	(3.4, 2.7, 1.4)	(1.9, 1.5, 0.8)
13	(6.6, 5.2, 2.5)	(5.0, 4.2, 2.2)	(2.8, 2.3, 1.6)
14	(5.8, 8.4, 2.1)	(4.2, 3.4, 1.8)	(2.4, 1.9, 1.2)
15	(4.8, 3.4, 1.6)	(3.2, 2.5, 1.3)	(1.8, 1.4, 0.7)

In Table 5, the error of BS-EKF at all test points was significantly lower than the other two models. PF showed significant errors in multiple tests, especially in complex motion trajectories, with significantly higher error values than other models. For example, for test points numbered 6 and 9, the errors of PF were (11.6, 6.2, 3.0) and (9.2, 4.8, 2.3), respectively, while the errors of BS-EKF were only (3.3, 2.8, 2.1) and (2.6, 2.1, 1.4). This indicated that PF was prone to significant prediction bias when dealing with high dynamics and complex movements. The EKF performance was between PF and BS-EKF. Although its error value was smaller than that of the PF model, it still exhibited significant errors at certain test points, such as test points numbered 2 and 8.

To more comprehensively verify the performance of the proposed BS-EKF model in different scenarios, the study introduces three models: Scaled-YOLOv4 [11], U-Net-based CNN [12], and FAST R-CNN +  FPN [20] as comparison models. The evaluation indicators include Average Trajectory Error (ATE), Trajectory Coverage Rate (TCR) and computational complexity (processing time and memory usage). The experimental results are shown in Table 6.

**Table 6:** Comprehensive Performance Comparison.

Dataset	Model	ATE/px	TCR/%	Processing time (ms/frame)	Memory usage (MB/frame)	References
TTNet	Scaled-YOLOv4	14.8	87.2	45.6	215.3	Hashmi MF et al. [11]
	U-Net based CNN	15.7	86.3	132.8	305.7	Yang Y et al. [12]
	FAST R-CNN + FPN	14.3	87.8	119.7	298.1	Rong Z [20]
	BS-EKF	12.4	90.1	66.3	152.4	This study
PingPongNet	Scaled-YOLOv4	21.3	80.6	52.4	241.5	Hashmi MF et al. [11]
	U-Net based CNN	22.5	79.4	136.2	310.8	Yang Y et al. [12]
	FAST R-CNN + FPN	20.7	81.7	133.1	317.8	Rong Z [20]
	BS-EKF	19.3	84.8	76.1	171.8	This study
SportsNet	Scaled-YOLOv4	31.2	71.4	58.7	256.2	Hashmi MF et al. [11]
	U-Net based CNN	32.4	70.3	140.9	330.6	Yang Y et al. [12]
	FAST R-CNN + FPN	30.5	72.7	145.3	340.1	Rong Z [20]
	BS-EKF	28.4	75.7	84.2	192.9	This study

From the perspective of key indicators, BS-EKF shows significant advantages in TCR. On the TTNet dataset, its TCR is 90.1%. In terms of computational complexity, its processing time is 66.3ms per frame, which is slightly higher than Scaled-YOLOv4’s 45.6ms per frame, reflecting the additional computational overhead caused by the complexity of the algorithm. In terms of space complexity, the memory footprint of BS-EKF is 171.8MB, which is lower than the memory footprint of U-Net-based CNN and FAST R-CNN+FPN. On PingPongNet, BS-EKF continues to lead in TCR, with TCR reaching 84.8%, which is higher than FAST R-CNN+FPN’s 81.7%. On the SportsNet data set, although the TCR of BS-EKF is better than other models, the computational complexity still needs to be balanced, especially in terms of processing speed, which is slightly inferior to YOLOv4. Overall, BS-EKF has advantages in detection performance and trajectory capture capabilities, and is suitable for practical application scenarios that require a balance between accuracy and resources. Finally, since PingPongNet contains challenging and complex scenes such as illumination changes and dynamic backgrounds, these scenes can effectively verify the robustness and adaptability of the model. Therefore, the performance of each model under illumination changes and dynamic background conditions is tested, and the results are shown in Table 7.

**Table 7:** Performance under Varying Lighting and Dynamic Background.

Dataset	Scenario condition	Model	mAP	ATE/px	TCR/%	References
PingPongNet	Varying lighting	Scaled-YOLOv4	0.84	22.5	79.4	Hashmi MF et al. [11]
		U-Net based CNN	0.83	23.8	78.3	Yang Y et al. [12]
		FAST R-CNN + FPN	0.85	21.7	80.1	Rong Z [20]
		BS-EKF	0.87	20.8	82.6	This study
	Dynamic background	Scaled-YOLOv4	0.83	21.5	78.1	Hashmi MF et al. [11]
		U-Net based CNN	0.81	23.7	76.5	Yang Y et al. [12]
		FAST R-CNN + FPN	0.84	21.3	78.8	Rong Z [20]
		BS-EKF	0.85	21.9	80.5	This study

In scenarios with changing illumination, BS-EKF had a mAP of 0.87 and a TCR of 82.6%, both higher than other models, and the lowest ATE of only 20.8px. The ATE of FASTR-CNN+FPN in this scenario was 21.7px, slightly inferior to BS-EKF, but its detection efficiency was excellent. In the scenario with dynamic background, FASTR-CNN+FPN had the lowest ATE of 21.3px, slightly better than BS-EKF’s 21.9px, but BS-EKF’s TCR reached 80.5%, ahead of Scaled-YOLOv4’s 78.1%. This result shows that BS-EKF is more robust overall in complex environments, while FASTR-CNN+FPN performs better in accurately locating target trajectories in specific scenarios.

## 4. Conclusion

To meet the needs of an electronic referee system for table tennis events that balances fairness and accuracy, this study used the background difference method to extract motion targets and combined extended KF to accurately estimate the trajectory. A table tennis motion target detection and estimation model with BS-EKF was designed. On the VOC2007 dataset, the mAP value of BS-EKF was 0.94, demonstrating high detection accuracy across 20 target categories, with detection accuracy of 0.99 for bird, aircraft, and train. Subsequently, YOLOv4, Faster R-CNN, SSD, and BS-EKF took an average of 61.0ms, 250.4ms, 50.6ms, and 103.9ms to detect different categories in the dataset. In the complete video image simulation test, BS-EKF only had deviation at the falling point, and its predicted trajectory was the most consistent with the real trajectory, with a small positional difference. The maximum difference in the horizontal and vertical directions was 10.7 and 4.3 pixels, respectively. In the testing of partially missing information in video images, BS-EKF also showed the highest trajectory prediction accuracy, with a maximum error of (3.3, 2.8, 2.1) in its three-dimensional coordinates, lower than the comparison model. In summary, the BS-EKF model shows significant advantages in dealing with complex backgrounds and missing targets. In addition, the proposed method has broad expansion potential. It can not only be used in the electronic referee system of table tennis events, but also be extended to other high-speed motion target detection scenarios, such as badminton and tennis events, as well as real-time target tracking and trajectory prediction tasks in industrial robots.

## 5. Limitation and furture work

Although the study has achieved good performance in target detection and trajectory prediction in table tennis, there are still some limitations and future development directions. The current method shows high detection accuracy under dynamic backgrounds, but its robustness in extreme lighting changes, complex backgrounds and severe occlusion scenes needs to be further improved. In addition, the experiment relies on high-performance computing equipment, and the complexity of the algorithm poses challenges to real-time and adaptability in resource-constrained environments. Future research can optimize computing efficiency by lightweighting the model and introduce deep learning methods to further improve detection accuracy and adaptability.

## Supporting information

S1 Data Minimal data set definition. (DOCX)

## References

[1] Wang Y, Sun Z, Luo Y, Zhang H, Zhang W, Dong K, et al. A novel trajectory-based ball spin estimation method for table tennis robot. IEEE Trans Ind Electron. 2024;71(8):9244–54. doi: 10.1109/tie.2023.3319743

[2] Mokayed H, Quan TZ, Alkhaled L, Sivakumar V. Real-time human detection and counting system using deep learning computer vision techniques. Artif Intell Appl. 2022;1(4):205–13. doi: 10.47852/bonviewaia2202391

[3] Zheng C, Lu M, Zeng Y, Hu M, Geng X, Xiao Y. The impact of wrist joint movement on stroke effect during topspin forehand in table tennis. Int J Perform Anal Sport. 2021;21(3):324–35. doi: 10.1080/24748668.2021.1885839

[4] Wang L, Zhou Z, Zou Q. Analysis system for table tennis techniques and tactics using data mining. Soft Comput. 2023;27(19):14269–84. doi: 10.1007/s00500-023-09082-z

[5] Liu W, Zhou Z, Shen Y, Zhang H. Stroke performance relevance model for elite table tennis matches. Int J Perform Anal Sport. 2022;22(4):558–70. doi: 10.1080/24748668.2022.2089514

[6] Ren W. A novel approach for automatic detection and identification of inappropriate postures and movements of table tennis players. Soft Comput. 2024;28(3):2245–69. doi: 10.1007/s00500-023-09587-7

[7] Shi Z, Jia Y, Shi G, Zhang K, Ji L, Wang D, et al. Design of motor skill recognition and hierarchical evaluation system for table tennis players. IEEE Sensors J. 2024;24(4):5303–15. doi: 10.1109/jsen.2023.3346880

[8] Yen C-T, Chen T-Y, Chen U-H, Wang G-C, Chen Z-X. Feature fusion-based deep learning network to recognize table tennis actions. Comput Mater Contin. 2023;74(1):83–99. doi: 10.32604/cmc.2023.032739

[9] Wang J, Wu J, Cao A, Zhou Z, Zhang H, Wu Y. Tac-miner: visual tactic mining for multiple table tennis matches. IEEE Trans Vis Comput Graph. 2021;27(6):2770–82. doi: 10.1109/TVCG.2021.3074576 33891553

[10] Abulwafa AE, Saleh AI, Saraya MS, Ali HA. A new ball detection strategy for enhancing the performance of ball bees based on fuzzy inference engine. Int J Intell Sys. 2021;37(11):9620–54. doi: 10.1002/int.22681

[11] Hashmi MF, Naik BT, Keskar AG. BDTA: events classification in table tennis sport using scaled-YOLOv4 framework. IFS. 2023;44(6):9671–84. doi: 10.3233/jifs-224300

[12] Yang Y, Kim D, Choi D. Ball tracking and trajectory prediction system for tennis robots. J Comput De Eng. 2023;10(3):1176–84. doi: 10.1093/jcde/qwad054

[13] Cruz N, Leiva F, Ruiz-del-Solar J. Deep learning applied to humanoid soccer robotics: playing without using any color information. Auton Robot. 2021;45(3):335–50. doi: 10.1007/s10514-021-09966-9

[14] Siratanita S, Chamnongthai K, Muneyasu M. A method of football-offside detection using multiple cameras for an automatic linesman assistance system. Wireless Pers Commun. 2019;118(3):1883–905. doi: 10.1007/s11277-019-06635-0

[15] Khan W, Ansell D, Kuru K, Amina M. Automated aircraft instrument reading using real time video analysis. 2016 IEEE 8th International Conference on Intelligent Systems (IS). 2016:416–20. doi: 10.1109/is.2016.7737454

[16] Faisal MM, Mohammed MS, Abduljabar AM, Abdulhussain SH, Mahmmod BM, Khan W, et al. Object Detection and Distance Measurement Using AI. 2021 14th International Conference on Developments in eSystems Engineering (DeSE). 2021:559–65. doi: 10.1109/dese54285.2021.9719469

[17] Yar H, Khan ZA, Ullah FUM, Ullah W, Baik SW. A modified YOLOv5 architecture for efficient fire detection in smart cities. Expert Syst Appl. 2023;231:120465. doi: 10.1016/j.eswa.2023.120465

[18] Zhao Y, Chen C, Sha J. Table tennis ball detection based on YOLOv5 and frameSORT. Fourth International Conference on Image Processing and Intelligent Control (IPIC 2024). 2024:76. doi: 10.1117/12.3038557

[19] Li H, Ali SG, Zhang J, Sheng B, Li P, Jung Y, et al. Video-based table tennis tracking and trajectory prediction using convolutional neural networks. Fractals. 2022;30(05):. doi: 10.1142/s0218348x22401569

[20] Rong Z. Optimization of table tennis target detection algorithm guided by multi-scale feature fusion of deep learning. Sci Rep. 2024;14(1):1401. doi: 10.1038/s41598-024-51865-3 38228726 PMC10792085

[21] Mavrogiannis P, Maglogiannis I. Amateur football analytics using computer vision. Neural Comput Appl. 2022;34(22):19639–54. doi: 10.1007/s00521-022-07692-6

[22] Akan S, Varlı S. Use of deep learning in soccer videos analysis: survey. Multimed Syst. 2022;29(3):897–915. doi: 10.1007/s00530-022-01027-0

[23] Houhou I, Zitouni A, Ruichek Y, Bekhouche SE, Kas M, Taleb-Ahmed A. RGBD deep multi-scale network for background subtraction. Int J Multimed Info Retr. 2022;11(3):395–407. doi: 10.1007/s13735-022-00232-x

[24] Delibaşoğlu İ. Moving object detection method with motion regions tracking in background subtraction. SIViP. 2023;17(5):2415–23. doi: 10.1007/s11760-022-02458-y

[25] Shao T, Luo Q. A sparse state kalman filter algorithm based on kalman gain. Circuits Syst Signal Process. 2022;42(4):2305–20. doi: 10.1007/s00034-022-02215-z

[26] Nair DS, Jagadanand G, George S. Torque estimation using kalman filter and extended kalman filter algorithms for a sensorless direct torque controlled BLDC motor drive: a Comparative Study. J Electr Eng Technol. 2021;16(5):2621–34. doi: 10.1007/s42835-021-00793-7

[27] Zhou JQ, Wei W. Research on detecting moving targets with an improved Kalman filter algorithm. KSII Trans Internet Inf Syst. 2023; 17(9): 2348–60. doi: 10.3837/tiis.2023.09.003

[28] Lyu X, Meng Z, Li C, Cai Z, Huang Y, Li X, et al. A dual adaptive unscented kalman filter algorithm for SINS-based integrated navigation system. J of Syst Eng Electron. 2024;35(3):732–40. doi: 10.23919/jsee.2024.000060

[29] Zhang X, Fan K, Ma W, Duan J, Liang J, Ji R. A novel fractional kalman filter algorithm with noisy input. IEEE Trans Circuits Syst II. 2023;70(3):1239–43. doi: 10.1109/tcsii.2022.3223945

[30] Huo Y, Yang K, Qi Y, Xu T. Robust maximum correlation entropy Kalman filtering algorithm based on S-functions under impulse noise. SIViP. 2024;18(S1):113–27. doi: 10.1007/s11760-024-03135-y

[31] Taghvaei A, Mehta PG. An optimal transport formulation of the ensemble kalman filter. IEEE Trans Autom Control. 2021;66(7):3052–67. doi: 10.1109/tac.2020.3015410

[32] Zhang X, Lin H, Liu G, He B. Distributed fuzzy extended kalman filter for multiagent systems. Int J Control Autom Syst. 2023;21(5):1692–703. doi: 10.1007/s12555-021-1060-6

[33] Jin Q, Yin Z, Dai Q. State observer design for microchannel cooling system using extended Kalman filter. Nume Heat Transf Part A Appl. 2022;84(5):433–48. doi: 10.1080/10407782.2022.2131658

[34] Jorgensen LT, Schou M, Stuart MB, Jensen JA. Tensor velocity imaging with motion correction. IEEE Trans Ultrason Ferroelectr Freq Control. 2021;68(5):1676–86. doi: 10.1109/TUFFC.2020.3046101 33347407

[35] Dai X, Lei Y, Roper J, Chen Y, Bradley JD, Curran WJ, et al. Deep learning-based motion tracking using ultrasound images. Med Phys. 2021;48(12):7747–56. doi: 10.1002/mp.15321 34724712 PMC11742242

