# Supplementary material for: Motion Object Detection Model for Electronic Referee Scoring in Table Tennis Events
Source: PLoS One. 2025 Mar 19;20(3):e0319558. doi: 10.1371/journal.pone.0319558 (PMC11922261; doi:10.1371/journal.pone.0319558)
Supplement: S1 Data — (DOCX) [file pone.0319558.s001.docx]

**No data in Figure 1**

**No data in Figure 2**

**No data in Figure 3**

**No data in Figure 4**

**No data in Figure 5**

**No data in Figure 6**

**The data in Figure 7**

| Epoch | Training set | | | |
| --- | --- | --- | --- | --- |
|  | YOLOv4 | Faster R-CNN | SSD | BS-EKF |
| 0 | 1.00 | 1.00 | 1.00 | 1.00 |
| 100 | 0.58 | 0.61 | 0.62 | 0.52 |
| 200 | 0.47 | 0.42 | 0.43 | 0.34 |
| 300 | 0.45 | 0.41 | 0.42 | 0.33 |
| 400 | 0.38 | 0.38 | 0.40 | 0.33 |
| 500 | 0.36 | 0.35 | 0.38 | 0.32 |
| Epoch | Test set | | | |
|  | YOLOv4 | Faster R-CNN | SSD | BS-EKF |
| 0 | 1.00 | 1.00 | 1.00 | 1.00 |
| 100 | 0.55 | 0.55 | 0.53 | 0.40 |
| 200 | 0.39 | 0.41 | 0.43 | 0.35 |
| 300 | 0.39 | 0.37 | 0.42 | 0.34 |
| 400 | 0.39 | 0.36 | 0.40 | 0.34 |
| 500 | 0.39 | 0.36 | 0.40 | 0.34 |

**The data in Figure 8**

| Recall | TTNet | | | |
| --- | --- | --- | --- | --- |
|  | YOLOv4 | Faster R-CNN | SSD | BS-EKF |
| 0.2 | 0.92 | 0.91 | 0.81 | 0.93 |
| 0.4 | 0.90 | 0.83 | 0.74 | 0.90 |
| 0.6 | 0.81 | 0.80 | 0.63 | 0.83 |
| 0.8 | 0.73 | 0.64 | 0.60 | 0.74 |
| 1.0 | 0.61 | 0.62 | 0.58 | 0.73 |
| Recall | PingPongNet | | | |
|  | YOLOv4 | Faster R-CNN | SSD | BS-EKF |
| 0.2 | 0.81 | 0.86 | 0.82 | 0.94 |
| 0.4 | 0.78 | 0.79 | 0.69 | 0.90 |
| 0.6 | 0.70 | 0.75 | 0.60 | 0.81 |
| 0.8 | 0.61 | 0.63 | 0.59 | 0.72 |
| 1.0 | 0.55 | 0.58 | 0.54 | 0.67 |
| Recall | SportsNet | | | |
|  | YOLOv4 | Faster R-CNN | SSD | BS-EKF |
| 0.2 | 0.80 | 0.84 | 0.81 | 0.88 |
| 0.4 | 0.75 | 0.72 | 0.74 | 0.78 |
| 0.6 | 0.64 | 0.67 | 0.63 | 0.73 |
| 0.8 | 0.58 | 0.62 | 0.57 | 0.68 |
| 1.0 | 0.52 | 0.56 | 0.51 | 0.63 |

**The data in Figure 9**

| Category | mAP | | | |
| --- | --- | --- | --- | --- |
|  | YOLOv4 | Faster R-CNN | SSD | BS-EKF |
| person | 0.95 | 0.93 | 0.92 | 0.97 |
| bird | 0.98 | 0.96 | 0.94 | 0.99 |
| cat | 0.98 | 0.94 | 0.90 | 0.98 |
| cow | 0.95 | 0.92 | 0.89 | 0.96 |
| dog | 0.98 | 0.95 | 0.92 | 0.98 |
| horse | 0.98 | 0.92 | 0.89 | 0.97 |
| sheep | 0.93 | 0.89 | 0.80 | 0.93 |
| aeroplane | 0.99 | 0.97 | 0.95 | 0.99 |
| bicycle | 0.98 | 0.95 | 0.93 | 0.98 |
| boat | 0.85 | 0.83 | 0.80 | 0.88 |
| bus | 0.98 | 0.94 | 0.92 | 0.98 |
| car | 0.95 | 0.93 | 0.91 | 0.97 |
| motorbike | 0.97 | 0.94 | 0.92 | 0.97 |
| train | 0.98 | 0.96 | 0.94 | 0.99 |
| bottle | 0.90 | 0.89 | 0.85 | 0.93 |
| chair | 0.85 | 0.82 | 0.80 | 0.90 |
| dining table | 0.73 | 0.69 | 0.65 | 0.80 |
| potted plant | 0.81 | 0.80 | 0.79 | 0.85 |
| sofa | 0.88 | 0.87 | 0.84 | 0.91 |
| tv monitor | 0.95 | 0.91 | 0.89 | 0.96 |

**The data in Figure 10**

| Horizontal axis (px) | True track | YOLOv4 | Faster R-CNN | SSD | BS-EKF |
| --- | --- | --- | --- | --- | --- |
| 0 | 600 | 600 | 600 | 600 | 600 |
| 100 | 283 | 268 | 275 | 243 | 264 |
| 200 | 423 | 431 | 401 | 434 | 425 |
| 300 | 96 | 148 | 142 | 234 | 121 |
| 400 | 301 | 303 | 276 | 420 | 287 |
| 500 | 172 | 206 | 231 | 270 | 197 |
| 600 | 247 | 249 | 195 | 272 | 198 |
| 700 | 134 | 164 | 149 | 120 | 142 |
| 800 | 224 | 220 | 221 | 241 | 226 |

**The data in Figure 11**

| Sampling time | YOLOv4 | |
| --- | --- | --- |
|  | Horizontal bias (px) | Vertical bias (px) |
| 1 | 0.8 | -13.7 |
| 5 | 2.3 | -14.7 |
| 10 | 4.1 | -7.4 |
| 15 | 8.7 | -17.2 |
| 20 | 0.2 | -17.5 |
| Sampling time | Faster R-CNN | |
|  | Horizontal bias (px) | Vertical bias (px) |
| 1 | 0.1 | -3.8 |
| 5 | 8.1 | -14.6 |
| 10 | 4.8 | -7.1 |
| 15 | 7.3 | -6.3 |
| 20 | 0.1 | -16.7 |
| Sampling time | SSD | |
|  | Horizontal bias (px) | Vertical bias (px) |
| 1 | 6.4 | -5.7 |
| 5 | 13.1 | -9.5 |
| 10 | 18.1 | -13.4 |
| 15 | 8.7 | -9.5 |
| 20 | 2.5 | -2.8 |
| Sampling time | BS-EKF | |
|  | Horizontal bias (px) | Vertical bias (px) |
| 1 | 0.0 | -3.2 |
| 5 | 3.8 | -3.8 |
| 10 | 3.9 | 2.0 |
| 15 | 6.0 | -2.4 |
| 20 | 0.0 | -4.1 |

**The data in Figure 12**

| Horizontal axis (px) | True track | PF (px) | EKF (px) | BS-EKF (px) |
| --- | --- | --- | --- | --- |
| 0 | 578 | 576 | 577 | 587 |
| 100 | 271 | 219 | 267 | 269 |
| 200 | 409 | 401 | 418 | 401 |
| 300 | 101 | 194 | 184 | 101 |
| 400 | 298 | 257 | 301 | 299 |
| 500 | 234 | 253 | 192 | 224 |
| 600 | 256 | 252 | 251 | 196 |
| 700 | 152 | 172 | 169 | 102 |
| 800 | 234 | 219 | 228 | 224 |
